# Laboratory Detection of Artemisinin-Resistant Plasmodium falciparum

**DOI:** 10.1128/AAC.01924-13

**Published:** 2014-06

**Authors:** Kesinee Chotivanich, Rupam Tripura, Debashish Das, Poravuth Yi, Nicholas P. J. Day, Sasithon Pukrittayakamee, Char Meng Chuor, Duong Socheat, Arjen M. Dondorp, Nicholas J. White

**Affiliations:** a Department of Clinical Tropical Medicine, Faculty of Tropical Medicine, Mahidol University, Bangkok, Thailand; b Mahidol-Oxford University Tropical Medicine Research Unit, Faculty of Tropical Medicine, Mahidol University, Bangkok, Thailand; c The National Center for Parasitology, Entomology, and Malaria Control, Phnom Penh, Cambodia; d Centre for Tropical Medicine, Churchill Hospital, Oxford, United Kingdom

## Abstract

Conventional 48-h *in vitro* susceptibility tests have low sensitivity in identifying artemisinin-resistant Plasmodium falciparum, defined phenotypically by low *in vivo* parasite clearance rates. We hypothesized originally that this discrepancy was explained by a loss of ring-stage susceptibility and so developed a simple field-adapted 24-h trophozoite maturation inhibition (TMI) assay focusing on the ring stage and compared it to the standard 48-h schizont maturation inhibition (WHO) test. In Pailin, western Cambodia, where artemisinin-resistant P. falciparum is prevalent, the TMI test mean (95% confidence interval) 50% inhibitory concentration (IC_50_) for artesunate was 6.8 (5.2 to 8.3) ng/ml compared with 1.5 (1.2 to 1.8) ng/ml for the standard 48-h WHO test (*P* = 0.001). TMI IC_50_s correlated significantly with the *in vivo* responses to artesunate (parasite clearance time [*r* = 0.44, *P* = 0.001] and parasite clearance half-life [*r* = 0.46, *P* = 0.001]), whereas the standard 48-h test values did not. On continuous culture of two resistant isolates, the artemisinin-resistant phenotype was lost after 6 weeks (IC_50_s fell from 10 and 12 ng/ml to 2.7 and 3 ng/ml, respectively). Slow parasite clearance in falciparum malaria in western Cambodia results from reduced ring-stage susceptibility.

## INTRODUCTION

Artemisinin-based combination therapy (ACT) is the first-line treatment of falciparum malaria recommended by the World Health Organization (1). The artemisinin derivatives provide rapid and effective reduction of parasite biomass and gametocyte carriage (2, 3), while the partner drugs in ACTs are eliminated much more slowly, providing mutual augmentation of efficacy and protection from resistance (4, 5, 6). Widespread deployment of ACTs is one of the main factors underlying recent reductions in global malaria morbidity and mortality (7, 8, 9). Unfortunately, reduced *in vivo* susceptibility to artemisinin derivatives, manifest by prolongation of parasite clearance times, has emerged in Cambodia (10, 11, 12, 13) and, more recently, on the northwestern border of Thailand (14). ACT efficacy in these areas is falling (15). Despite obvious resistance *in vivo*, conventional 48-h *in vitro* tests have not shown corresponding reductions in *in vitro* susceptibility (13), and so they are not useful as an epidemiological tool for monitoring artemisinin resistance. We surmised that a reduction in susceptibility in the ring-stage, without a corresponding reduction in the susceptibility in the more mature parasite stages, could explain this apparent discrepancy (13, 16). We therefore developed a simple adaptation of the standard WHO *in vitro* 48-h antimalarial drug susceptibility test, focusing on the ring-stage development of the parasite, and assessed its utility as a predictor of artemisinin resistance *in vivo*.

## MATERIALS AND METHODS

### Patients.

Plasmodium falciparum isolates were collected from adult patients enrolled in clinical trials conducted between 2009 and 2010 in the Pailin Referral Hospital in western Cambodia. Written informed consent was obtained from patients with no history of previous antimalarial treatment before enrollment in studies and collection of blood samples. These studies were approved by the Ministry of Health, Cambodia, and the Ethics Committee of the Faculty of Tropical Medicine, Mahidol University, Thailand, and are described in detail elsewhere (8, 17). Patients received artesunate (ATS) either 6 mg/kg of body weight or 8 mg/kg per day for the first 3 days of treatment before receiving mefloquine 15 mg/kg on day 3 and 10 mg/kg on day 4. Venous blood samples (2 mL) for parasite culture were collected into sterile sodium heparin tubes before treatment.

### Parasite clearance.

Thick and thin blood films were prepared using standard procedures. Parasite species, morphology, and parasitemia were assessed by light microscope examination to determine the proportions of parasites at various developmental stages. Parasite staging was determined as described previously (18). Parasite species identification was confirmed by nested PCR (19). Parasite counts were taken every six hours until clearance. Parasite clearance was measured as the time until the first of two successive negative parasite counts, and the parasite clearance half-life was derived from the slope of the log-linear segment of the parasite clearance profile and was calculated as described previously (20).

### *Ex vivo* susceptibility assay.

Artesunate (Guilin Pharmaceutical Co., Ltd., China) was prepared as 60 mg/ml of stock solution dissolved in 1 ml of 5% NaHCO_3_, kept at −30°C, and used within 1 month of preparation. Artesunate (ATS) was further diluted in RMPI-1640 culture medium to 1 mg/ml (stored at 4°C and used within 1 week of preparation), and then by serial 2-fold dilutions to obtain final concentrations ranging from 0.01 to 400 ng/ml of ATS. The first row of the 96-well drug plate contained no drug.

The susceptibility of P. falciparum to artesunate was evaluated at 24 h (trophozoite maturation inhibition [TMI]) and 48 h (schizont maturation inhibition, WHO standard test). P. falciparum-infected blood was centrifuged at 800 × *g* at 4°C for 5 min. After the plasma and buffy coat were removed, the packed red cells were washed three times in RPMI 1640 and resuspended to make a 3% cell suspension in the complete medium. A 75-μl cell suspension was added to the predosed antimalarial microtiter plate. Each drug concentration was tested in duplicate. Wells without drugs were included as controls. After addition of the cell suspension, thorough mixing was performed to dissolve the drug, and then the lid was placed over the plate. The samples were incubated at 37°C in 5% CO_2_ for 24 and 48 h for the TMI and WHO assays, respectively. At the end of the incubation, thick and thin blood films were made from each well. The standard laboratory P. falciparum Thai strain (TM267) was used as an internal control. In order to check the stability of the susceptibility of the artemisinin resistance phenotype, four parasite isolates (2 sensitive [ANL1 and -3] and 2 resistant [ANL3 and -4]) determined by *in vivo* clinical responses (parasite clearance time [PCT] and parasite clearance half-life) were cultured continuously in the absence of drugs *in vitro* at 3 to 5% parasitemia and 5% hematocrit. Blood films were made daily to check the culture parasitemias. The TMI susceptibility test was performed once each week.

### Evaluation of antimalarial drug susceptibility and data analysis.

Drug activity was expressed as the percentage of inhibition of maturation compared to control (no drug). For the 24-h TMI assay, the number of trophozoites (age 24 to 30 h) was counted per 100 parasitized red cells. Trophozoites were identified by morphology, size, nuclear/cytoplasm ratios, and presence of visible pigment (18). For the standard WHO 48-h assay, the number of schizonts (≥8 merozoites/schizont) per 100 infected red cells was counted. The 50% inhibitory concentration (IC_50_) was defined as the drug concentration providing 50% inhibition of the parasite development from ring stage to trophozoite (TMI, 24-h assay) or from ring stage to schizont (WHO, 48-h assay) compared to the parasite in the drug-free control wells. The IC_50_ was determined by sigmoid curve fitting using WinNonlin computer software (version 3.1; Pharsight Corporation, USA). Correlations were assessed by the method of Spearman. Statistical significance within and between groups was determined by the nonparametric Kruskal-Wallis or Mann Whitney U tests. All statistical analyses were performed using SPSS software.

## RESULTS

### Clinical correlates.

Between 2009 and 2010, *ex vivo* drug susceptibility assays were performed satisfactorily on 71 P. falciparum field isolates obtained from patients with malaria in Pailin, western Cambodia. At the start of drug exposure, ring-stage parasites with estimated ages of 6 to 16 h were the predominant stages in the patients' blood samples. Only 2 out of 71 cases presented with a mixture of late ring stages and early trophozoites. All isolates showed development to trophozoites in the control wells, all 71 (100%) IC_50_ values were obtained from the TMI tests and 56 (78%) from the WHO tests. The remaining 15 parasite samples were not evaluated satisfactorily as they developed into gametocytes at 48 h and so did not provide reliable curves.

**FIG 1:**
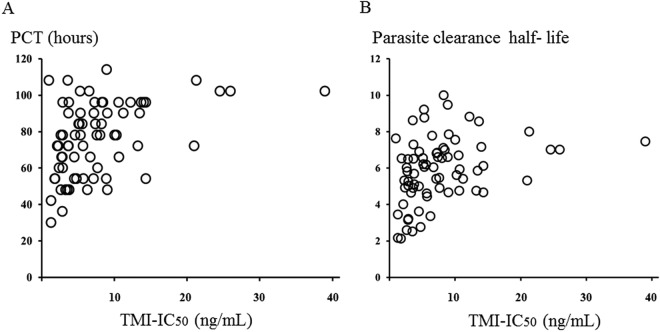
Correlation between 50% inhibitory concentration (IC_50_) from TMI test and *in vivo* responses. (A) IC_50_ plotted against parasite clearance time (PCT) for the first of two consecutive negative blood films; (B) IC_50_ plotted against parasite clearance half-life.

**FIG 2:**
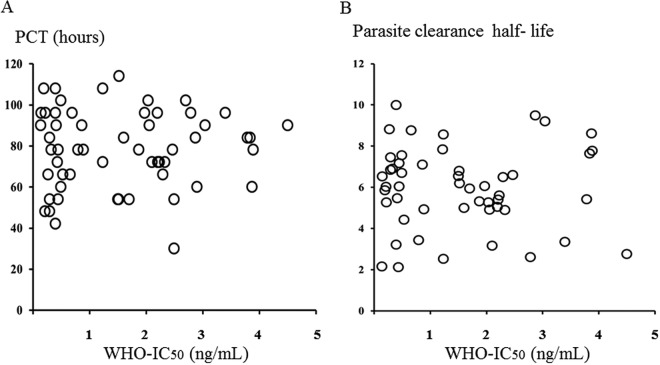
Correlation between 50% inhibitory concentration (IC_50_) from WHO test and *in vivo* responses. (A) IC_50_ plotted against parasite clearance time (PCT) for the first of two consecutive negative blood films; (B) IC_50_ plotted against parasite clearance half-life.

Complete inhibition of trophozoite and schizont maturation occurred in the wells containing 400 ng/ml of artesunate for all isolates evaluated. In Pailin, the mean (95% confidence interval [CI]) 50% inhibitory concentration (IC_50_) values for artesunate were 6.8 (5.2 to 8.3) ng/ml by the TMI test and 1.5 (1.2 to 1.8) ng/ml by the WHO test (Table 1) (*P* = 0.001). The IC_50_ of the reference strain (TM267) was 0.7 (0.5 to 0.9) ng/ml by TMI test and 0.9 (0.5 to 1.3) ng/ml by the 48-h WHO test. The TMI IC_50s_ correlated significantly with parasite clearance times (*r_s_* = 0.44, *P* = 0.001) and parasite clearance half-life (*r_s_* = 0.46, *P* = 0.001), whereas there were no significant correlations between IC_50_s from the WHO test and *in vivo* parasite clearance (*r_s_* = 0.2, *P* > 0.05) (Fig. 1 and 2). The sensitivity and specificity (95% CI) of a TMI of >8 ng/ml for identifying an infection with a parasite clearance half-life of >5.5 h were 32% (28 to 42%) and 72% (41 to 100%).

**TABLE 1:** IC_50_s of P. falciparum in Pailin, western Cambodia

Isolate origin (no. of isolates)	Mean (95% CI) IC_50_ (ng/ml) for:	
	24-h TMI test	48-h WHO test
Pailin, Cambodia (71)	6.8 (5.2–8.3)^*a*^	1.5 (1.2–1.8)
Thai laboratory strain (TM267) (1)	0.7 (0.5–0.9)	0.9 (0.5–1.3)

a Significant at a *P* value of <0.001.

### Phenotype stability.

**FIG 3:**
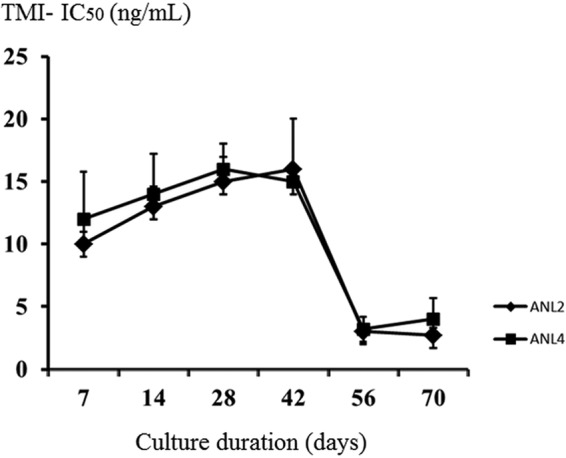
Stability of resistant phenotype. Data are presented as means and standard deviations (SD).

The mean (SD) IC_50_s of sensitive parasites (ANL1 and -3) assessed by the TMI test did not change significantly over 6 weeks of *in vitro* cultivation (3.3 [0.83] for ANL1 and 4.3 [0.89] for ANL3), whereas the IC_50_s of resistant parasites (ANL2 and -4) decreased significantly. The mean (SD) IC_50_s fell from 10 to 2.7 ng/ml for ANL2 and from 12 to 4 ng/ml for ANL4 (Fig. 3). Further investigations on these isolates are ongoing.

## DISCUSSION

The *ex vivo* assessment of antimalarial drug susceptibility is a valuable tool in tracking and monitoring antimalarial drug resistance. *Ex vivo* susceptibility measurement allows assessment of the response of the parasites in the absence of host factors which might influence drug efficacy *in vivo*. Several techniques, including DNA-based and antigen-based methods, have been used in addition to standard microscopy in the schizont inhibition maturation assay (WHO). DNA-based techniques include measurement of parasite uptake of tritium-labeled hypoxanthine (21) or Sybr green staining of parasite DNA (22). Antigen-based techniques include the histidine-rich protein 2 assay (23) and double-site lactate dehydrogenase antigen capture enzyme-linked immunosorbent assay (ELISA) methods (24). Current methods all evaluate the efficacy of the drug after 48 to 72 h of incubation, which reflects the activity of the drug on stages of parasite development from rings to mature schizonts. Most or all of the signal derives from the second half of the asexual life cycle, so any changes which affect predominantly the first half are largely obscured.

In this study, conducted in the epicenter of artemisinin resistance, we confirmed that standard *in vitro* testing does not identify artemisinin-resistant P. falciparum, whereas a simple modified 24-h assay assessing trophozoite maturation inhibition does. This TMI assay correlated significantly with the key clinical measures of parasite clearance time and parasite clearance half-life. This finding is consistent with a recent report that ring stages of P. falciparum parasites from western Cambodia show increased survival after 6-h pulse exposures to 700 nM dihydroartemisinin (25). In contrast, ring stages at 2 to 4 h postinvasion were reported to exhibit hypersensitivity to a short pulse of artemisinin derivative exposure (26). In addition, the artemisinin resistance phenotype was characterized by the ability to recover from drug-induced dormancy following exposure to high concentrations of the drug (27). All these tests point to a common mechanism underlying artemisinin resistance-reduced ring-stage susceptibility, although they examine different aspects and thus give different values. A parasite clearance half-life of >5.5 h has been used as a cutoff for identifying artemisinin resistance. The predictive value of the TMI in identifying such infections was moderate, with a value of >8 ng/ml having sensitivity and specificity values of 32% and 72%, respectively. Whether this represents a limitation of the method or intrinsic variation remains to be seen. In this area of Cambodia, most parasites are artemisinin resistant, so variance in parasite clearance results more from other factors. It is possible that *in vivo*/*in vitro* correlations, and thus the predictive value, of the TMI test would be better in areas where artemisinin-resistant parasites do not predominate (and so drug susceptibility is a greater contributor to overall variance in parasite clearance). One particular limitation of the test (and any others focusing only on the ring stage) is that it relies on patients having a relatively synchronous young population of parasites at presentation, but some patients present with parasites at different stages of development, and this results in different ring-stage drug exposures. Differences in the parasite age at the start of the drug exposure can therefore have a significant impact on the IC_50_ of these ring stage tests (26). The TMI technique is conceptually simple but it is laborious and therefore slow, and it requires microscopy experience or special training. High-throughput enzyme-linked immunosorbent assay (ELISA)-based methods based on ring-stage-specific antigens are under development.

While there is clear evidence for heritability in artemisinin resistance, the phenotype appears unstable, at least in the two parasites we studied in continuous culture (14, 28). This has also been observed in laboratory studies in which artemisinin-resistant P. falciparum was first selected, although it has also been possible to eventually select for stably resistant lines. Epigenetic processes may contribute to the phenotype. This suggests that the resistance phenotype may provide a substantial fitness disadvantage and so could be readily lost, but as the genetic predisposition has been acquired it seems likely that resistance would return rapidly on further selection, although this remains to be established.

## References

[1] World Health Organization. 2010 Guidelines for the treatment of malaria, 2nd ed, p 13–47 WC 770. World Health Organization Press, Geneva, Switzerland

[2] Hien TT White NJ 1993 Qinghaosu. Lancet 341:603–608. 10.1016/0140-6736(93)90362-K 8094838

[3] White NJ 2008 Qinghaosu (artemisinin): the price of success. Science 320:330–334. 10.1126/science.1155165 18420924

[4] Ashley EA White NJ 2005 Artemisinin-based combinations. Curr. Opin. Infect. Dis. 18:531–536. 10.1097/01.qco.0000186848.46417.6c 16258328

[5] Nosten F White NJ 2007 Artemisinin-based combination treatment of falciparum malaria. Am. J. Trop. Med. Hyg. 77(6 Suppl):181–192 18165491

[6] White NJ 2008 The role of anti-malarial drugs in eliminating malaria. Malar. J. 7(Suppl 1):S8. 10.1186/1475-2875-7-S1-S8 19091042 PMC2604872

[7] Barnes KI White NJ 2005 Population biology and antimalarial resistance: the transmission of antimalarial drug resistance in Plasmodium falciparum. Acta Trop. 94:230–240. 10.1016/j.actatropica.2005.04.014 15878154

[8] Dondorp AM Fanello CI Hendriksen IC Gomes E Seni A Chhaganlal KD Bojang K Olaosebikan R Anunobi N Maitland K Kivaya E Agbenyega T Nguah SB Evans Gesase JS Kahabuka C Mtove G Nadjm B Deen J Mwanga-Amumpaire J Nansumba Karema MC Umulisa N Uwimana A Mokuolu OA Adedoyin OT Johnson WB Tshefu Onyamboko AKMA Sakulthaew T Ngum WP Silamut K Stepniewska K Woodrow CJ Bethell D Wills B Oneko M Peto TE von Seidlein L Day NP White NJ; AQUAMAT group 2010 Artesunate versus quinine in the treatment of severe falciparum malaria in African children (AQUAMAT): an open-label, randomised trial. Lancet 376:1647–1657. 10.1016/S0140-6736(10)61924-1 21062666 PMC3033534

[9] World Health Organization. 2011 World malaria report 2011. World Health Organization, Geneva, Switzerland http://www.who.int/malaria/world_malaria_report_2011/en

[10] Alker AP Lim P Sem R Shah NK Yi P Bouth DM Tsuyuoka R Maguire JD Fandeur T Ariey F Wongsrichanalai C Meshnick SR 2007 Pfmdr1 and *in vivo* resistance to artesunate-mefloquine in falciparum malaria on the Cambodian-Thai border. Am. J. Trop. Med. Hyg. 76:641–647 17426163

[11] Denis MB Tsuyuoka R Lim P Lindegardh N Yi PTop Socheat SND Fandeur T Annerberg A Christophel EM Ringwald P 2006 Efficacy of artemether-lumefantrine for the treatment of uncomplicated falciparum malaria in northwest Cambodia. Trop. Med. Int. Health 11:1800–1807. 10.1111/j.1365-3156.2006.01739.x 17176344

[12] Noedl H Se Y Schaecher K Smith BL Socheat D Fukuda MM; Artemisinin resistance in Cambodia 1 (ARC1) Study Consortium. 2008 Evidence of artemisinin-resistant malaria in western Cambodia. N. Engl. J. Med. 359:2619–2620. 10.1056/NEJMc0805011 19064625

[13] Dondorp AM Nosten F Yi P Das D Phyo AP Tarning J Lwin KM Ariey F Hanpithakpong W Lee SJ Ringwald P Silamut K Imwong M Chotivanich K Lim P Herdman T An SS Yeung S Singhasivanon P Day NP Lindegardh N Socheat D White NJ 2009 Artemisinin resistance in Plasmodium falciparum malaria. N. Engl. J. Med. 361:455–467. 10.1056/NEJMoa0808859 19641202 PMC3495232

[14] Phyo AP Nkhoma S Stepniewska K Ashley EA Nair S McGready R ler Moo C Al-Saai S Dondorp AM Lwin KM Singhasivanon P Day NP White NJ Anderson TJ Nosten F 2012 Emergence of artemisinin-resistant malaria on the western border of Thailand: a longitudinal study. Lancet 379:1960–1966. 10.1016/S0140-6736(12)60484-X 22484134 PMC3525980

[15] Carrara VI Zwang J Ashley EA Price RN Stepniewska K Barends M Brockman Anderson AT McGready R Phaiphun L Proux S van Vugt M Hutagalung R Lwin Phyo KMAP Preechapornkul P Imwong M Pukrittayakamee S Singhasivanon P White NJ Nosten F 2009 Changes in the treatment responses to artesunate-mefloquine on the northwestern border of Thailand during 13 years of continuous deployment. PLoS One 4:e4551. 10.1371/journal.pone.0004551 19234601 PMC2641001

[16] Saralamba S Pan-Ngum W Maude RJ Lee SJ Tarning J Lindegårdh N Chotivanich K Nosten F Day NP Socheat D White NJ Dondorp AM White LJ 2011 Intrahost modeling of artemisinin resistance in Plasmodium falciparum. Proc. Natl. Acad. Sci. U. S. A. 108:397–402. 10.1073/pnas.1006113108 21173254 PMC3017155

[17] Das D Tripura R Phyo AP Lwin KM Tarning J Lee SJ Hanpithakpong W Stepniewska K Menard D Ringwald P Silamut K Imwong M Chotivanich K Yi P Day NP Lindegardh N Socheat D Nguon C White NJ Nosten F Dondorp AM 2013 Effect of high-dose or split-dose artesunate on parasite clearance in artemisinin-resistant falciparum malaria. Clin. Infect. Dis. 56:e48–e58. 10.1093/cid/cis958 23175556 PMC3563392

[18] Silamut K White NJ 1993 Relation of the stage of parasite development in the peripheral blood to prognosis in severe falciparum malaria. Trans. R. Soc. Trop. Med. Hyg. 87:436–443. 10.1016/0035-9203(93)90028-O 8249075

[19] Snounou G Viriyakosol S Zhu XP Jarra W Pinheiro L do Rosario VE Thaithong S Brown KN 1993 High sensitivity of detection of human malaria parasites by the use of nested polymerase chain reaction. Mol. Biochem. Parasitol. 61:315–320. 10.1016/0166-6851(93)90077-B 8264734

[20] Flegg JA Guerin PJ White NJ Stepniewska K 2011 Standardizing the measurement of parasite clearance in falciparum malaria: the parasite clearance estimator. Malar. J. 10:339. 10.1186/1475-2875-10-339 22074219 PMC3305913

[21] Webster HK Boudreau EF Pavanand K Yongvanitchit K Pang LW 1985 Antimalarial drug susceptibility testing of Plasmodium falciparum in Thailand using a microdilution radioisotope method. Am. J. Trop. Med. Hyg. 34:228–235 3885770 10.4269/ajtmh.1985.34.228

[22] Bacon DJ Latour C Lucas C Colina O Ringwald P Picot S 2007 Comparison of a SYBR green I-based assay with a histidine-rich protein II enzyme-linked immunosorbent assay for in vitro antimalarial drug efficacy testing and application to clinical isolates. Antimicrob. Agents Chemother. 51:1172–1178. 10.1128/AAC.01313-06 17220424 PMC1855478

[23] Noedl H Attlmayr B Wernsdorfer WH Kollaritsch H Miller RS 2004 A histidine-rich protein 2-based malaria drug sensitivity assay for field use. Am. J. Trop. Med. Hyg. 71:711–714 15642959

[24] Druilhe P Moreno A Blanc C Brasseur PH Jacquier P 2001 A colorimetric *in vitro* drug sensitivity assay for Plasmodium falciparum based on a highly sensitive double-site lactate dehydrogenase antigen-capture enzyme-linked immunosorbent assay. Am. J. Trop. Med. Hyg. 64:233–241 11463109 10.4269/ajtmh.2001.64.233

[25] Witkowski B Khim N Chim P Kim S Ke S Kloeung N Chy S Duong S Leang R Ringwald P Dondorp AM Tripura R Benoit-Vical F Berry A Gorgette O Ariey F Barale JC Mercereau-Puijalon O Menard D 2013 Reduced artemisinin susceptibility of Plasmodium falciparum ring stages in western Cambodia. Antimicrob. Agents Chemother. 57:914–923. 10.1128/AAC.01868-12 23208708 PMC3553720

[26] Klonis N Xie SC McCaw JM Crespo-Ortiz MP Zaloumis SG Simpson JA Tilley L 2013 Altered temporal response of malaria parasites determines differential sensitivity to artemisinin. Proc. Natl. Acad. Sci. U. S. A. 26:5157–5162. 10.1073/pnas.1217452110 23431146 PMC3612604

[27] Tucker MS Mutka T Sparks K Patel J Kyle DE 2012 Phenotypic and genotypic analysis of in vitro-selected artemisinin-resistant progeny of Plasmodium falciparum. Antimicrob. Agents Chemother. 56:302–314. 10.1128/AAC.05540-11 22083467 PMC3256069

[28] Anderson TJ Nair S Nkhoma S Williams JT Imwong M Yi P Socheat D Das D Chotivanich K Day NP White NJ Dondorp AM 2010 High heritability of malaria parasite clearance rate indicates a genetic basis for artemisinin resistance in western Cambodia. J. Infect. Dis. 201:1326–1330. 10.1086/651562 20350192 PMC2853733

